# No difference in persistence to treatment with atazanavir or darunavir in HIV patients in a real-world setting

**DOI:** 10.7448/IAS.17.4.19538

**Published:** 2014-11-02

**Authors:** Amanda M Farr, Stephen S Johnston, Corey Ritchings, Matthew Brouillette, Lisa Rosenblatt

**Affiliations:** 1 Life Sciences, Truven Health Analytics, Cambridge, MA, USA; 2 Life Sciences, Truven Health Analytics, Bethesda, MD, USA; 3 US Medical HIV, Bristol-Myers Squibb, Princeton, NJ, USA; 4 Health Economics and Outcomes Research, Bristol-Myers Squibb, Plainsboro, NJ, USA

## Abstract

**Introduction:**

There is a lack of data comparing the protease inhibitors (PIs) atazanavir (ATV) and darunavir (DRV) in a real-world setting. This study compared persistence (time to switch/discontinuation) to therapy between ATV-treated and DRV-treated patients with human immunodeficiency virus (HIV).

**Materials and Methods:**

Retrospective, observational cohort study using US insurance claims for commercially and Medicaid-insured patients. Patients were aged ≥18 years and initiated an ATV- or DRV-based regimen boosted with ritonavir between 7/1/2006 and 3/31/2013, with ≥6 months of continuous enrolment prior to and ≥3 months of continuous enrolment following initiation; patients were required to have ≥1 inpatient or outpatient medical claim with an ICD-9-CM diagnosis code for HIV during that time period of enrolment. Patients with no claims for antiretroviral therapy (ART) any time prior to initiation were considered to be ART-naïve. Time to switch/discontinuation was defined as the number of days from initiation of the regimen until earliest of: (1) a ≥30-day continuous gap in therapy in ATV or DRV; (2) a prescription claim for an ART agent that was not part of the initial regimen (with the exception of changes in concomitant nucleoside reverse transcriptase inhibitors or the addition of integrase inhibitors); (3) censoring at a ≥30-day continuous gap in therapy in ritonavir; (4) censoring at disenrolment from insurance benefits or (5) censoring at the study end date (9/30/2013 in the commercial data and 12/31/2013 in the Medicaid data). Time to switch/discontinuation was compared using incidence rates and multivariable Cox proportional hazards models adjusted for calendar time, patient demographics and clinical characteristics.

**Results:**

Table 1 displays the study results and cohort sample sizes. Mean ages across the cohorts were 41–42 years. The proportions of patients who were ART-naïve were 58–59% among the ATV/r cohorts and 53–55% among the DRV/r cohorts. There were no significant differences in the adjusted hazards of switch/discontinuation between the cohorts.

**Conclusions:**

The incidence of switch/discontinuation was higher among Medicaid patients (who may be socioeconomically disadvantaged) than Commercial patients. There were no significant differences in persistence (time to switch/discontinuation) with the initiated PI among HIV patients who initiated an ATV-based regimen versus a DRV-based regimen.

**Table 1:** Incidence and adjusted hazard ratio of switch/discontinuation among patients with HIV initiating an ATV- or DRV-based ART regimen

	Incidence of switch/discontinuation per 100 person-years (95% confidence interval)	Adjusted hazard ratio of switch/discontinuation atazanavir vs darunavir (95% confidence interval)	p-value for adjusted hazard ratio
Commercially insured patients: ART-naïve			
ATV (N = 1581)	48.3 (45.3–51.5)	1.119 (0.966–1.295)	0.134
DRV (N = 859)	47.1 (42.8–51.8)	Reference	
Commercially insured patients: ART-experienced			
ATV (*N*=1151)	42.7 (39.5–46.1)	1.091 (0.961–1.238)	0.177
DRV (N = 712)	39.2 (35.3–43.6)	Reference	
Medicaid-insured patients: ART-naïve			
ATV (N = 1276)	90.0 (84.4–96.1)	1.120 (0.965–1.300)	0.135
DRV (N = 419)	87.3 (77.4–98.5)	Reference	
Medicaid-insured patients: ART-experienced			
ATV (N = 895)	84.9 (78.4–91.8)	0.929 (0.779–1.109)	0.416
DRV (N = 377)	84.2 (74.3–95.5)	Reference	

ATV = atazanavir; DRV = darunavir; ART = antiretroviral therapy.

